# Effect of Repeated Ramadan Fasting in the Hottest Months of the Year on Renal Graft Function

**DOI:** 10.5812/numonthly.14362

**Published:** 2014-03-01

**Authors:** Fayez Hejaili, Salim Qurashi, Salih Binsalih, Maha Jaradt, Abdulla Al Sayyari

**Affiliations:** 1 King Saud bin Abdulaziz University for Health Sciences, Riyadh, Saudi Arabia; 2 King Abdulaziz Medical City, Riyadh, Saudi Arabia

**Keywords:** Fasting, Kidney, Glomerular Filtration Rate

## Abstract

**Background:**

Adult Moslems are required to fast during the lunar month of Ramadan every year. Although the sick and travelers, as well as some other specified groups, are exempted from this requirement.

**Objectives:**

To investigate the effect of repeated Ramadan fasting during the hottest months of the year on renal graft functions.

**Patients and Methods:**

This was a prospective cohort study comparing two groups of renal transplant receivers; one group had fasted for two consecutive Ramadan months during 2011 and 2012, while the other group had not fasted. The baseline eGFR (estimated glomerular filtration rate) was compared to the eGFR carried out 19.6 ± 1.3 months later, within and between groups. Further subgroup analysis was done according to eGFR baseline; low (< 45 mL/min/1.73 m^2^), moderate 45-75 (mL/min/1.73 m^2^), and high (> 75 mL/min/1.73 m^2^).

**Results:**

There were 43 fasting and 37 non-fasting participants with comparable; ages, gender, type of transplant, and baseline eGFR and serum creatinine (SCr). The fasting participants, however, had a longer elapsed time since their transplantation. In the fasting group, SCr and eGFR did not change from baseline after a mean follow-up period of 19.6 ± 1.3 months; SCr of 105.1 ± 55.4 and 114.2 ± 71.5 µmol/L, respectively (P-value = 0.8), and eGFR 75.6 ± 29.2 and 70.2 ± 28.1 mL/min/1.73 m^2^, respectively (P-value = 0.09). Similarly, no significant changes were observed in the non-fasting group; Sr of 123.1 ± 67 and 115.8 ± 65.2 µmol/L, respectively (P-value = 0.6), and eGFR of 65.9 ± 25.9 and 68.8 ± 24.6 mL/min/1.73 m^2^, respectively (P-value = 0.6). On subgroup analysis, according to the eGFR level, we found no significant differences in the eGFR, before and after 19.6 ± 1.3 months, in the severe and moderate subgroups. However, a significant but similar drop was noted in the high GFR subgroups in both the fasting subgroup (96.4 ± 15 to 84.9 ± 20.7 mL/min/1.73 m^2^; P = 0.17) and in the non-fasting subgroup (92.9 ± 15.8 to 82.3 ± 18.2 mL/min/1.73 m^2^; P = 0.019).

**Conclusions:**

Fasting in the month of Ramadan in two consecutive years, and during the hottest months, in Riyadh, Saudi Arabia, did not adversely affect kidney graft function.

## 1. Background

Adult Moslems are mandated to fast during the lunar month of Ramadan every year. The sick and travelers, as well as some other specified groups, are exempted from this duty. There have been a number of reports, including some from our center, concerning the effect of Ramadan fasting on kidney graft function. In general, these patients have shown no adverse effect from fasting in Ramadan. However, it should be noted that in all of these previous reports, Ramadan occurred during the cooler months of the year. Moreover, the graft function in patients studied in those reports was favorable (1-7).

## 2. Objectives

In this present study, we look at the effect of fasting in two consecutive Ramadan months (in 2011 and 2012), during the hottest months of the year in Riyadh, Saudi Arabia, which is one of the hottest cities in the world. Moreover, the cohort study included patients with stages 3b and 4 chronic kidney diseases (CKD).

## 3. Patients and Methods

This was a prospective cohort study carried out at King Abdulaziz Medical City in Riyadh, Saudi Arabia. The glomerular filtration rate (GFR) was estimated using a four-variable modification of diet in renal disease (MDRD) formula (8), in patients with renal transplantation who had fasted, and in a control group who had not. The decision to fast was left to the patient after consulting with them about any possible risk from fasting on their graft function. The month of Ramadan coincided with August in the Gregorian calendar in 2011, and from July 20 to August 18, in 2012. Forty-three patients who had voluntarily fasted during both consecutive Ramadan months were included and their results were compared with 37 patients who had not fasted. The eGFR was calculated within a month before Ramadan of 2011 and 19.6 ± 1.3 months after it. During both Ramadan months, the daily hours that the fasting patients remained without food or drink ranged from 12 to 14 hours. The highest temperature during the day was 49°C (9), while the average humidity ranged from 12% to 14% (10). The fasting participants acted as their own control group for detecting the impact of fasting on allograft function. In addition, a control group composed of non-fasting participants was enrolled in the study. The purpose of having a non-fasting control group was to assess whether any change in renal function in the fasting patients was related to fasting per se or to progression of renal impairment in renal allografts due to the hot weather.

The patients were advised to drink at least 2.5 L of fluid after breaking their fast, to take the morning dose of immunosuppressives at the time of breaking the fast, and to take the evening dose just before the start of the fast. Patients with diabetes mellitus took their anti-diabetic medication during the evening and the pattern of eating and treatment was reversed from day to night. IRB approval was obtained for the research.

### 3.1. Statistical Analyses

Baseline characteristics were summarized by calculating the percentages (for categorical data) and means and standard deviations (for continuous data). A chi-square test and t-test were used to assess the differences between the fasting and non-fasting participants in terms of baseline characteristics. Differences in eGFR were calculated. Paired t-tests and a Wilcoxon signed rank sum test were used to assess the differences between the pre- and post-Ramadan eGFR among the fasting participants.

## 4. Results

There were 43 and 37 fasting and non-fasting participants, respectively, with mean ages of 45.2 ± 15.6 and 43.3 ± 15.4 years, respectively (P = 0.6). The time elapsed since transplantation in the two groups were 64.4 (30.4) and 27.7 (36.7) months, respectively (P = 0.0001) (Table 1). The mean serum creatinine levels before Ramadan were similar in the fasting group (105.9 ± 55.4 and 123.1 ± 67 µmol/L, respectively, with P-value = 0.2), as were the mean eGFRs (75.6 ± 29.2 and 65.9 ± 25.9 mL/min/1.73 m^2^, respectively, with P-value = 0.1). In the fasting group, mean baseline serum creatinine levels, and levels after a mean follow-up period of 19.6 ± 1.3 months after Ramadan, were similar (105.8 ± 55.6 and 114.2 ± 71.5 µmol/L, respectively, with P value = 0.8). Similarly, we noted no significant change in the eGFR over the same period in this group (75.6 ± 29.2 and 70.2 ± 28.1 mL/min/1.73 m^2^, respectively, with P = 0.09) (Table 2). The mean baseline serum creatinine level and the mean level after a mean follow-up period of 20 ± 1.3 months after Ramadan in the non-fasting group were similar (123.1 ± 67 and 115.8 ± 65.2.5 µmol/L, respectively, with P-value = 0.6). Moreover, we noted no significant changes in the eGFR over the same period in this group (65.9 ± 25.9 and 68.8 ± 24.6. mL/min/1.73 m^2^, respectively, with P-value = 0.6) (Table 2). 

**Table 1.** Comparing Baseline Characteristics in the Fasting and Non-fasting Groups ^a, b^

	Fasting, n = 43	Non-fasting, n = 37	P value
**Age, y**	45.2 ± 15.6	43.3 ± 15.4	0.6
**Male, %**	46.5	56.8	0.4
**Transplant type: living, %**	62.8	70.3	0.5
**Duration after transplant, mo**	65.9 ± 30.2	29.2 ± 36.7	
**Baseline pre-Ramadan SCr, umol/L**	105.9 ± 55.4	123.1 ± 67	0.2
**Baseline pre-Ramadan eGFR, mL/min/1.73 m^2^**	75.6 ± 29.2	65.9 ± 25.9	0.1
**Mean period between Ramadan to last SCr/eGFR, mo**	19.6 ± 1.3	20 ± 1.3	0.27

^a^ Abbreviations: Scr, serum creatinine; eGFR, estimated glomerular filtration rate.

^b^ Data are presented as mean ± SD.

**Table 2.** Comparing Changes in Renal Function Between Baseline Values and at the End of Follow-up in the Fasting and Non-fasting Groups ^a, b^

	Baseline, mL/min/1.73 m^2^	Final (after 18 months), mL/min/1.73 m^2^	P value
**eGFR**			
Fasting group	75.6 ± 29.2	70.2 ± 28.1	0.09
Non-fasting group	65.9 ± 25.9	68.8 ± 24.6	0.6
**SCr**			
Fasting group	105.1 ± 55.4	114.2 ± 71.5	0.8
Non-fasting group	123.1 ± 67	115.8 ± 65.2	0.6

^a^ Abbreviations: Scr, serum creatinine; eGFR, estimated glomerular filtration rate.

^b^ Data are presented as mean ± SD.

We subdivided the fasting group into three subgroups, according to the degree of renal function at baseline, including; low (< 45 mL/min/1.73 m^2^), moderate (45-75 mL/min/1.73 m^2^), and high GFR (> 75 mL/min/1.73 m^2^). We found no significant differences in the eGFR before and after 19.6 ± 1.3 months in the fasting group, in the severe and moderate subgroups. However, a significant drop was noted in the high eGFR subgroup in both the fasting subgroup (96.4 ± 15 to 84.9 ± 20.7 mL/min/1.73 m^2^; P value = 0.17) and the non-fasting subgroup (92.9 ± 15.8 to 82.3 ± 18.2 mL/min/1.73 m^2^; P value = 0.019) (Table 3). 

**Table 3.** Results According to the Renal Function at Baseline in the Two Groups ^a, b^

	Baseline eGFR	eGFR at 18 months	P value
**Fasting group**			
Low baseline eGFR < 45, mL/min/1.73 m^2^, n = 8	30.1 ± 10.7	30.4 ± 12.5	0.9
Moderate baseline eGFR 45-75, mL/min/1.73 m^2^, n = 11	63.2 ± 10.3	66.9 ± 20.4	0.6
High baseline eGFR > 75, mL/min/1.73 m^2^, n = 24	96.4 ± 15	84.9 ± 20.7	0.017
**Non-fasting group**			
Low baseline eGFR < 45, mL/min/1.73 m^2^, n = 7	29.2 ± 11.1	29.8 ± 13.3	0.3
Moderate baseline eGFR 45-75, mL/min/1.73 m^2^, n = 17	60.5 ± 7.9	63.2 ± 19.1	0.6
High baseline eGFR > 75, mL/min/1.73 m^2^, n = 13	92.9 ± 15.8	82.3 ± 18.2	0.019

^a^ Abbreviation: eGFR, estimated glomerular filtration rate.

^b^ Data are presented as mean ± SD.

## 5. Discussion

Fasting during the lunar month of Ramadan is a mandatory requirement for all healthy adult Moslems. However, the sick, travelers, debilitated elderly people, and pregnant and lactating women, are exempt from this obligation (11). Those for whom fasting may be detrimental to their health, are also exempted from fasting (11). Ramadan fasting lasts from sunrise to sunset during which time the person refrains completely from eating or drinking. Ramadan is the ninth month of the Moslem lunar year, which is eleven days shorter than the Gregorian calendar (solar year). As such, Ramadan time moves throughout the four seasons and makes a full circle every 33 years. The last time Ramadan months coincided with the hottest month (August) in Riyadh was, therefore, back in 1980. This was the year in which the first renal transplantation was carried out in Saudi Arabia. Now, thirty three years later, we have 12 550 patients with functioning kidney transplants in Saudi Arabia (12). There are now many Moslem countries performing organ transplants, with thousands of Moslems living with a transplanted organ.

Numerous studies have previously assessed the effect of Ramadan fasting on patients with renal transplantation, or with normal or moderately impaired renal function, and they found no adverse effects (1-7). There were no significant differences between the fasters and the non-fasters with regard to the changes in GFR, MAP, and urinary protein excretion, between baseline and the third Ramadan (2). In a previous cohort study of 33 kidney transplant recipients in our center, we found no negative effects following fasting during three consecutive Ramadan months. In this study, we found no changes in the estimated GFR after fasting for three consecutive Ramadan months, even after adjusting for; age, presence of diabetes mellitus, baseline GFR, proteinuria, or the elapsed time from transplantation. The results in this group were compared to a group of non-fasting participants and no differences were detected between these two groups (2). However, comparing our previous study with this study, the three consecutive Ramadan months occurred in October and November when the weather was distinctly cooler in Riyadh, and the fasting times were shorter. In a cohort of 17 fasting post-transplant patients, we found no significant changes in serum electrolytes, serum creatinine, or the fractional excretion of sodium (FENa). However, in that study, the patients were followed for only one week after the end of a single Ramadan and their mean elapsed time after transplantation was only two years; while Ramadan again, coincided with the cooler months in that year (1). In a cohort study from Iran on 41 fasting patients, no negative impact was seen on graft function following fasting during Ramadan. However, the weather associated with Ramadan in that study was much cooler than in our study. Moreover, the effect of Ramadan on renal function in that study was assessed just after the end of Ramadan. In that study they had a subgroup of fasting participants with eGFR < 60 mL/min (mean 47.8 ± 11.2 mL/min) in whom, they reported no significant change in eGFR following fasting (49.7 ± 12.7 mL/min) (6). In our study, we found that fasting during two consecutive Ramadan months in one of the hottest countries in the world does not adversely affect graft function even after a follow-up period of over 19 months. With regard to the subgroup analysis, we found no adverse effects from fasting during two consecutive Ramadan months, in patients with moderate and severe degrees of low eGFR.
